# Bone Fracture-Treatment Method: Fixing 3D-Printed Polycaprolactone Scaffolds with Hydrogel Type Bone-Derived Extracellular Matrix and β-Tricalcium Phosphate as an Osteogenic Promoter

**DOI:** 10.3390/ijms22169084

**Published:** 2021-08-23

**Authors:** Seokhwan Yun, Dami Choi, Dong-Jin Choi, Songwan Jin, Won-Soo Yun, Jung-Bo Huh, Jin-Hyung Shim

**Affiliations:** 1 Department of Mechanical Engineering, Korea Polytechnic University, Siheung-si 15073, Korea; yuntobi@kpu.ac.kr (S.Y.); seaottersarecute@protonmail.com (D.-J.C.); songwan@kpu.ac.kr (S.J.); 2 Research Institute, T&R Biofab Co., Ltd., Siheung-si 15073, Korea; choidm@tnrbiofab.com; 3 Department of Prosthodontics, Dental Research Institute, Dental and Life Sciences Institute, School of Dentistry, Pusan National University, Yangsan-si 50612, Korea

**Keywords:** β-tricalcium phosphate, bone-derived extracellular matrix, bone fracture, polycaprolactone, 3D printing

## Abstract

Bone formation and growth are crucial for treating bone fractures. Improving bone-reconstruction methods using autologous bone and synthetic implants can reduce the recovery time. Here, we investigated three treatments using two different materials, a bone-derived decellularized extracellular matrix (bdECM) and β-tricalcium phosphate (β-TCP), individually and in combination, as osteogenic promoter between bone and 3D-printed polycaprolactone scaffold (6-mm diameter) in rat calvarial defects (8-mm critical diameter). The materials were tested with a human pre-osteoblast cell line (MG63) to determine the effects of the osteogenic promoter on bone formation in vitro. A polycaprolactone (PCL) scaffold with a porous structure was placed at the center of the in vivo rat calvarial defects. The gap between the defective bone and PCL scaffold was filled with each material. Animals were sacrificed four weeks post-implantation, and skull samples were preserved for analysis. The preserved samples were scanned by micro-computed tomography and analyzed histologically to examine the clinical benefits of the materials. The bdECM–β-TCP mixture showed faster bone formation and a lower inflammatory response in the rats. Therefore, our results imply that a bdECM–β-TCP mixture is an ideal osteogenic promoter for treating fractures.

## 1. Introduction

Osteogenesis is crucial for treating bone fractures (such as avulsion, comminuted, and crush fractures) and bone diseases such as osteoporosis [1]. Although minor bone fractures can recover without surgical intervention, the recovery period can be long and inconvenient [1]. For multiple complex fractures, recovery is not guaranteed, and most cases require invasive surgery [1,2]. Autologous bone and synthetic implants have been adopted to accelerate the healing time, guarantee recovery, and reduce the need for surgical intervention [3,4,5,6,7,8]. Although patient-derived autologous bones show low immunogenicity, there is limited supply of that bone. Allogenic materials exhibit similar limitations. Synthetic implants provide a defined composition and can be designed using immune-free materials [9]. The above-described grafting materials show low integration to the original bone, causing improper non-union problems. Decellularized extracellular matrix (dECM) provides a native cellular environment, combining the original tissue-specific composition and architecture [10,11]. During decellularization, cells mediating immune rejection are removed. Therefore, both allogenic and heterogenic tissue-derived dECM are suitable [10]. They can be obtained from animals and provide cues to endogenous stem cells for homing, optimizing proliferation, and maintaining stemness. Furthermore, dECM can be used as biomimetic bioink in three-dimensional (3D)-printing systems [12,13,14]. ECM are easily solubilized with appropriate viscosity, facilitating their use as bioink materials in 3D-printing systems.

The chemical composition of β-tricalcium phosphate (β-TCP) resembles that of bone minerals [5], and β-TCP shows excellent osteoconductive properties [15,16]. Polycaprolactone is extensively used as a bioresorbable polymer and provides a good platform for designing fabricated implants requiring long-term degradation kinetics in bone-tissue engineering [17]. Calcium phosphate can be applied in granular or powdered form. However, the granular form is brittle and powdery, and causes inflammation due to size-matter toxicity [18].

**Figure 1:**
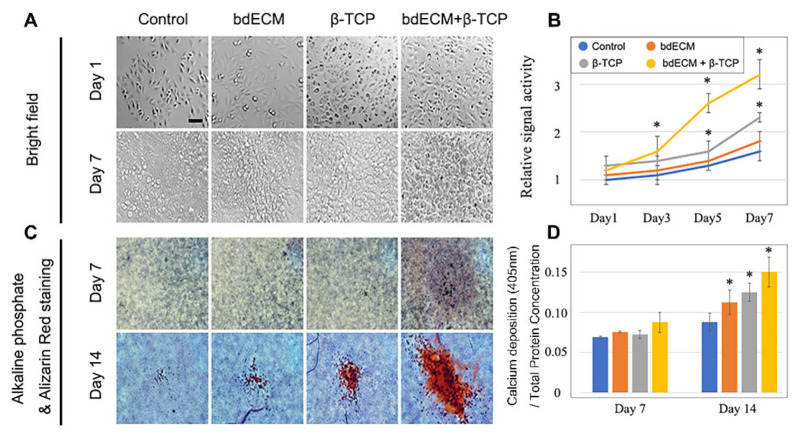
Effects of three different materials on the pre-osteoblast cell line MG63 (**A**,**B**). Cell number was increased in the group of bdECM + β-TCP compared to control in Days 3, 5, and 7; (**C**). Bone formation was also increased in the group of bdECM + β-TCP compared to control in Day 14; (**D**). Calcium deposition was measured and normalized by cellular protein content. Similar to the bone formation, calcium deposition was also increased in the group of bdECM + β-TCP compared to control in Day14. Asterisk indicates significant difference (*p* < 0.05) compared to control. Scale bar indicated 20 µm.

Here, a slurry-like material was implanted by mixing powder-type TCP with soluble bone-derived ECM (bdECM) and evaluated as a material for inducing native bone–graft fusion. We tested three different materials as osteogenic promoters (bdECM, β-TCP, and a combination of both) and evaluated their properties with MG63 cells in vitro. In vivo, a 3D-printed PCL scaffold (6-mm diameter) was fabricated, and three different materials were used to fill the PCL scaffold–bone defect gap using a rodent calvarial-defect model (Figure 1). This versatile model enabled evaluation of bone formation in living organisms within a reproducible, non-load-bearing orthotopic site. Macrophage distributions in the surrounding tissue were measured to confirm the inflammatory responses. The mixed material showed accelerated bone formation and reduced macrophage distribution [19,20].

## 2. Results

### 2.1. Human Osteoblast Proliferation and Differentiation

Cell proliferation increased and was faster in Group 3 than the other groups. By Day 3 and thereafter, cell number was most increased in Group 3. By Days 5 and 7, 2.08-fold and 2.46-fold more cells were observed compared to the control, respectively (Figure 1A,B). The cells showed the greatest maturation in Group 3 (Figure 1C). Alkaline phosphatase (ALP) staining revealed 1.88-fold, 1.24-fold, and 1.1-fold greater differentiation in Group 3 versus the control group, Group 1, and Group 2, respectively (Figure 1D).

### 2.2. Osteogenic Intracellular-Sginaling Pathway

**Figure 2:**
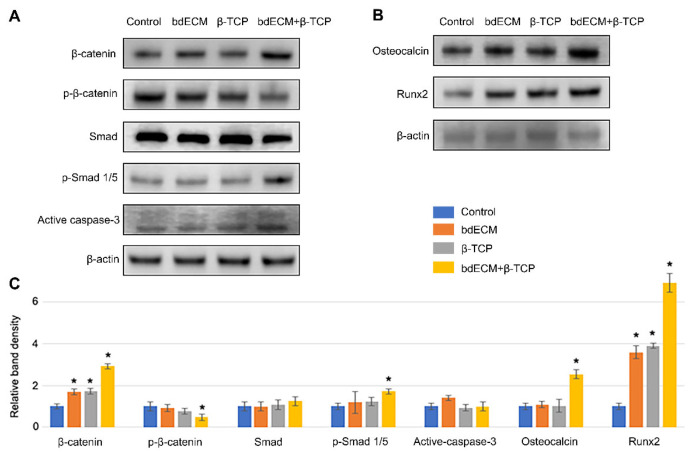
Western blotting analysis for intracellular signaling pathway and osteogenic differentiation. (**A**). Protein derived from pre-osteoblast cell line MG63 was blotted for alteration of signaling pathway in Day 2. β-catenin and phospho-β-catenin were measured to confirm canonical Wnt signaling pathway. Smad and phospho-Smad1/5 were measured to confirm BMP2 signaling pathway. Active caspase3 was measure for cytotoxicity and showed no difference; (**B**). Protein derived from pre-osteoblast cell line MG63 was blotted for bone formation marker osteocalcin and Runx2 in Day 5; (**C**). Band densities were measured at least three times for statistical analysis and all data were expressed as mean ± standard deviation. Asterisk indicates significant difference (*p* < 0.05) compared to control.

Western blotting was performed on Day 2 post-cell seeding to evaluate intracellular signaling and osteogenic differentiation. β-catenin expression increased 3.02-fold in Group 3 (versus the control), and phospho-β-catenin levels decreased 0.81-fold (Figure 2A,C). Phospho-Smad (p-Smad)1/5 levels increased in Group 3 (1.82-fold versus control; Figure 2A,C), confirming the involvement of bone-morphogenetic protein (BMP) signaling. Bone-formation markers (osteocalcin and Runx2) increased by Day 5 (2.72-fold and 6.90-fold, respectively, versus Group 1), consistent with the ALP-staining results (Figure 1C and Figure 2B,C). Cleaved-caspase 3 was not significant enhanced in any group (Figure 2A,C).

### 2.3. Bone-Regeneration Effects in the Rodent Model

**Figure 3:**
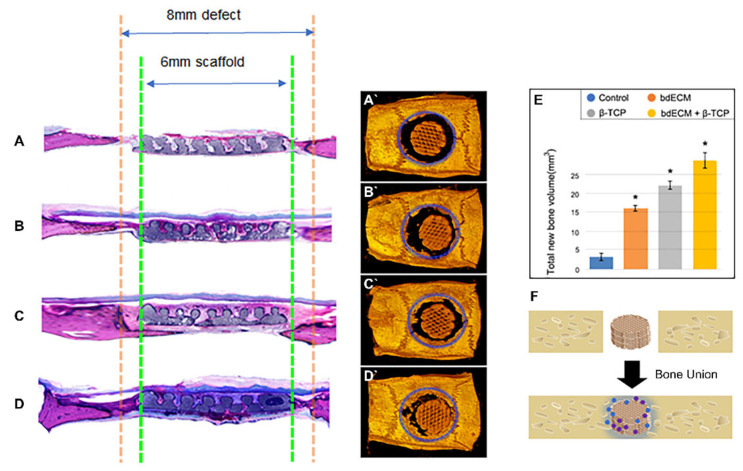
Masson’s trichrome staining and computed tomographic scan. (**A**–**D**). Green line indicate scaffold boundary and orange line indicate bone defect. Soft tissues were observed in all groups (red staining), blue staining that indicates newly formed bone tissue was observed in the group of bdECM + β-TCP; (**A`**–**D`**). Blue dotted line indicates calvarial defect edge, and the bone area inside the blue dotted line is the new bone. According to CT images, the new bone volume in the group of bdECM + β-TCP increased compared to other groups. Blue arrow indicates newly formed bone originated from host bone tissue and black arrow indicates bone union of scaffold and host bone tissue(**D`**); (**E**). All data were expressed as mean ± standard deviation and asterisk indicates statistical significance (*p* < 0.05) compared to control; (**F**). Schematic illustration of bone union between host and graft by bdECM and β-TCP.

**Figure 4:**
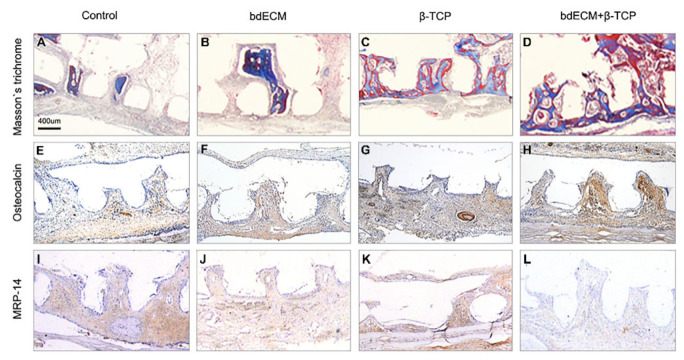
Histological analysis of animal defect area. (**A**–**D**). Masson’s trichrome staining showed blue staining on the cross-section of the scaffold. A strong blue positive area was most frequently observed in the mixed material (bdECM + β-TCP) (**D**); (**E**–**H**). In osteocalcin immunohistochemistry analysis, brown positive cells were most frequently observed in the group of bdECM + β-TCP (**H**); (**I**–**L**). MRP-14 immunohistochemistry showed unusual macrophage cell distribution, with bdECM and mixture groups showing reduced macrophage distribution (**L**).

Computed tomography (CT) scanning revealed bone formation in the calvarial-defect rodent model. Bone and PCL scaffolds (but not the promoter material) were visible in the CT-scanning images. Thus, the area of newly formed bone was calculated by subtracting the scaffold area from the defect areas (within the blue-dotted circles; Figure 3A–D). Group 3 showed a significant bone-regeneration effect, versus the other groups. Groups 1, 2, and 3 showed 5.36-fold (2.98 ± 0.68 mm^3^ versus 15.83 ± 0.86 mm^3^), 7.37-fold (2.98 ± 0.68 mm^3^ versus 21.95 ± 1.39 mm^3^), and 9.66-fold (2.98 ± 0.68 mm^3^ versus 28.78 ± 1.88 mm^3^) increased volumes of newly formed bones versus the control (Figure 3E). The host bone connected with the grafted scaffold (Figure 3D, black arrows), and grew towards it (Figure 3D, blue arrows). Masson’s trichrome staining (blue) indicated new bone formation. Similar to the CT results, Masson’s trichrome staining also revealed the effect of bone formation by the bdECM–β-TCP mixture (Figure 3A–D). In the control group, only soft tissue was observed in the gap between the host bone and grafted scaffold (Figure 3A). In Group 3, newly formed bone tissue was observed in the gap (Figure 3D). Masson’s trichrome staining and osteocalcin immunostaining showed increased bone formation in Group 3 versus the other groups (Figure 4A–H). The newly formed bone tissue integrated the grafted scaffold, especially in Group 3 (Figure 4D,H). Osteocalcin expression was assessed for bone formation, and myeloid-related protein-14 (MRP-14) was assessed for macrophages (Figure 4E–L).

### 2.4. Effect of β-TCP and bdECM in Interleukin (IL)-6 Production

**Figure 5:**
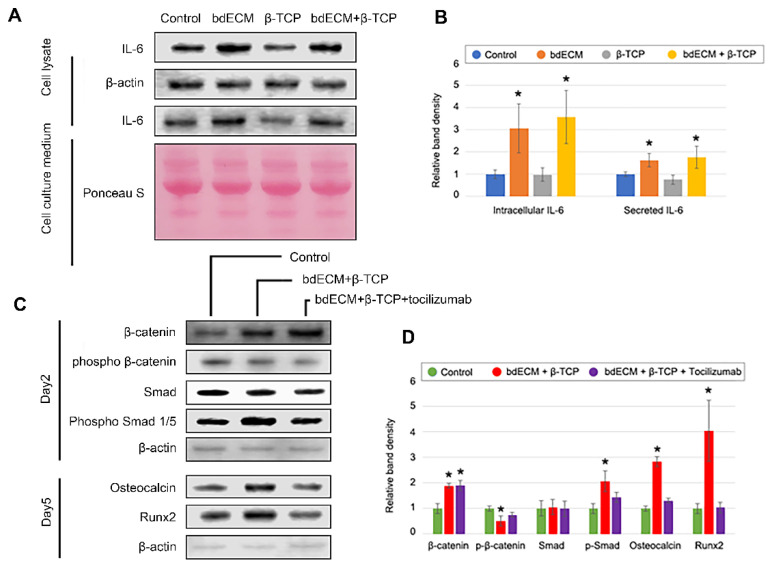
Loss of function study of IL-6. (**A**,**B**). Three different materials provided with pre-osteoblast MG63 cell lysate and cell culture medium-derived protein were blotted. The bdECM group and mixture group (bdECM and β-TCP) showed IL-6 overexpression compared to the control and β-TCP group both in cell lysate and cell culture medium; (**C**,**D**). The IL-6 antagonist tocilizumab-treated mixture group showed neutralization of the BMP2 signaling pathway and bone formation. In contrast, the canonical Wnt signaling pathway was not neutralized by tocilizumab. All data were expressed as mean ± standard deviation and asterisk indicates statistical significance (*p* < 0.05) compared to control.

In the absence of an anti-inflammatory agent, inflammatory responses were reduced in the bdECM and combination-treatment group (Figure 4I–L). MRP 14 immunohistochemistry showed no anti-inflammatory effects were observed for bdECM (Figure 4J,K). Several inflammatory and anti-inflammatory cytokines were evaluated in the materials -treated MG-63 cells. Most cytokines (except IL-6) were undetectable or showed extremely low concentrations in both enzyme-linked immunosorbent assays (not shown) and dot blots (Supplementary Figure S1). Western blotting of culture media and cell lysates showed that IL-6 levels increased significantly (3.06-fold and 3.56-fold, respectively, versus the control; Figure 5A,B). In addition, the bdECM group was treated with TCZ (an IL-6 antagonist), and MG63 cells were treated with β-TCP. TCZ did not affect the levels of β-catenin or *p*-β-catenin, which are key regulators of the Wnt-signaling pathway that neutralize the effect of BMP2 signaling by altering *p*-Smad expression levels. Furthermore, osteocalcin and Runx2 levels decreased in the TCZ-treated group (Figure 5C,D).

Although bdECM did not contain IL-6 (Supplementary Figure S1), it increased IL-6 production by MG63 cells.

## 3. Discussion

Osteogenesis is the most important process for repairing bone fractures. We hypothesized that bdECM induces both IL-6 and BMP2 to promote early osteogenesis and that β-TCP provides calcium after early osteogenesis. β-TCP is chemically similar to human bone and has bone-induction properties [21]. However, the microenvironment of bone tissue is composed of many proteins [22] that are difficult to mimic artificially; thus, actual tissue is preferable. bdECM was chosen as the best candidate; however, essential elements (including calcium and phosphorus) are lost during bdECM production. These lost elements are provided by β-TCP. Therefore, a combination of β-TCP and bdECM is suitable. MG63 cells showed the highest maturation levels in Group 3 (Figure 1). β-catenin was upregulated in Group 3 versus Groups 1 and 2 (Figure 2). Smad1/5 (key molecules in the BMP-signaling pathway) were also upregulated. The Wnt- and BMP-signaling pathways appeared to have synergistic effects and can be triggered by biochemical stimulation. Aqueous β-TCP alters cell-signaling pathways in the osteoblast cell line, MC3T3 [22]. Proteomic analysis of bdECM revealed that bdECM components trigger biochemical-signaling pathways, including BMP2 and Wnt3a [22,23]. Based on its viscosity (Supplementary Figure S2C), bdECM is expected to function as an adhesive, in addition to its biological properties. bdECM has a high collagen content, so its viscosity increases in vivo at 37 °C, enabling its performance as a sticky glue.

PCL is the most economical and accessible material in the 3D-bioprinting field and has been used in implant materials. It is structurally supportive of defective and damaged areas and slowly degrades after recovery (Supplementary Figure S2D). Thus, we designed the PCL scaffold to match the bone defect, but with a smaller size. The gap between the bone defect and PCL scaffold was filled with the three different materials as promoters, and the in vitro bone-formation effect was greatest in Group 3.

CT images confirmed that bone regeneration started at the native bone (Figure 3). Since the PCL scaffold itself does not contain stem cells or growth factors, it is difficult to say that it is osteoinductive [24]. However, the bdECM+TCP material induced bone regeneration from native bone and connected to the scaffold. This suggests that the bdECM+TCP material was osteoconductive and that it clearly induced osteogenesis between the scaffold and native bone, which was confirmed by Masson’s Trichrome staining. To quantify bone induction by bdECM+TCP, bone union between the newly created bone and the scaffold and the native bone must be evaluated using a method, such as three-point bending, to study the recovery area. Thus, a limitation of this study is that only the scaffold strength was evaluated, and further research is required (Supplementary Figure S2).

Inflammatory cells showed the lowest distribution in the grafted region of Group 3. No anti-inflammatory effects have been reported for the promoters or PCL. Inflammatory cells migrate through damage-associated molecular pattern-mediated chemoattraction. Large molecular differences are observed between wounded and healthy conditions. It was predicted that bdECM would provide a healthy microenvironment in the defect area to reduce inflammatory cytokines (i.e., IL-1, IL-12, IL-18, TNF-α, IFN-γ, and granulocyte-M-CSF) and activate anti-inflammatory cytokines (i.e., IL-1RA, IL-4, IL-6, IL-10, IL-11, IL-13, and TGF-β) [25]. IL-10 and TGF-β are secreted by macrophages [26]. IL-6 elicits anti-inflammatory effects and is secreted by osteo-lineage cells, including MG63 cells [27,28]. Additionally, IL-6 can promote bone formation [26,27,28]. IL-6 induces BMP-2 expression and reduces inflammation [27], suggesting its role in regulating BMP-2-induced and inflammation-modulated bone regeneration. Thus, treatment with the bdECM–β-TCP mixture activated IL-6, the BMP-2-signaling pathway, and the Wnt-signaling pathway to exert therapeutic effects (Figure 5, Supplementary Figure S1). We did not confirm which components of bdECM increased the IL-6 expression; however, loss-of-function analysis indicated its contribution to IL-6 production.

## 4. Materials and Methods

### 4.1. PCL Scaffold Fabrication

Polycaprolactone (molecular weight, 65,000; Sigma-Aldrich, St. Louis, MO, USA) was dispensed from a steel syringe of 3D printing head for 45 min at 110 °C, with a pneumatic pressure of 550 kPa [18]. A 3D printer with four different dispensing heads was used to prepare the 3D-PCL scaffold using a layer-by-layer process, with a 3D printer (T&R Biofab Co., Ltd., Siheung, Korea) and in-house code generation software. To design the model, SolidWorks computer-aided design (CAD) software (Dassault Systemes, Vélizy-Villacoublay, France) was used. Chip-type PCL was placed in a 10-mL steel syringe with a steel nozzle (500-μm diameter). The syringe and nozzle were loaded in a controllable head.

The triangular PCL scaffold pore structure was designed with a 300-μm line width, a 300-μm pore size (50% porosity), and a 400-μm height with four layers (each 100 μm thick). The PCL scaffold was designed in a circular shape and a 6-mm diameter.

### 4.2. Preparation of the bdECM–β-TCP Mixture

An enzymatic-decellularization protocol was used [20]. bdECM powder (T&R Biofab Co., Ltd., Siheung, Korea) was solubilized (digested) in 0.5 M acetic acid containing 100 mg pepsin (Sigma-Aldrich)/g bdECM at 4 °C for 72 h. To create a neutral bdECM solution (pH 7.4), bdECM solution (pH 3.0) was mixed with 10× α-minimum essential medium (α-MEM)(Sigma-Aldrich, CA, USA) and resuspension buffer (2.2 g NaHCO_3_ (Sigma-Aldrich), 4.77 g HEPES (Sigma-Aldrich), 17.0 g NaOH (Sigma-Aldrich) in 100 mL distilled water) at a volume ratio of 8:1:1. The β-TCP power (Sigma-Aldrich) was added to solubilized bdECM solution at a final concentration of 0.18 g/scaffold.

### 4.3. Pre-Osteoblast Culture, Proliferation, and Differentiation Measurements

MG63 cells were obtained from the American Type Culture Collection (ATCC, Manassas, VA, USA) and maintained in a humidified incubator (37 °C, 5% CO_2_) with α-MEM (Sigma-Aldrich) containing 10% fetal bovine serum. Cells (1 × 10^6^) were seeded into a 100-mm diameter cell culture plate and maintained for 3–4 days, after which 1 × 10^5^ cells were seeded onto each scaffold in a 96-multi-well white plate and later counted using a Luminescent Cell Viability Assay Kit (Promega, Madison, WI, USA). To evaluate cell differentiation, 1 × 10^5^ cells were seeded in a 24-well cell culture plate and calcium deposition was measured. Bone differentiation was evaluated by Titrate-Resistant Acid Phosphatase and ALP Double-Staining Kit (Takara, Shiga, Japan). ALP activities were determined by absorbance measurements at 405 nm, with a microplate reader (Bio-Tek instruments, Winooski, VT, USA) and normalized to the protein concentration [29].

### 4.4. Western Blotting

Total cellular protein samples were collected from each group. Western blotting was performed following the guideline supplied by Bio-Rad (Hercules, CA, USA). Primary antibodies (Table S1) were diluted 1:1000 in 5% skimmed milk (BD Biosciences, Franklin Lakes, NJ, USA).

### 4.5. Rat Calvarial Defect Model

Sprague–Dawley rats were purchased (Orient Bio, Seoul, Korea). All animals were housed in a pathogen-free (SPF) facility under a 12-h light and dark cycle with ad libitum feeding. The animals were randomly assigned to four groups: control group (n = 3), dECM-scaffold group (Group 1; n = 8), β-TCP-scaffold group (Group 2; n = 8), and dECM plus β-TCP mixture-scaffold group (Group 3; n = 8). Animals were anesthetized with 2.5% isoflurane (Hanapharma, Busan, Korea) mixed with oxygen, and the skull was exposed. On the base of the skull bregma or skull lambda, a cylindrical bone defect (8-mm diameter) was created using a round burr drill bit. The study was performed at the Laboratory Animal Resource Center of Pusan National University and approved by the Pusan National University Institutional Animal Care and Use Committee (PNU-2016-1407).

### 4.6. Micro-CT and Histologic Assessment

Harvested tissue samples were fixed in 10% formaldehyde before μCT scanning (Skyscan-1173, v1.6, Bruker-CT, Kontich, Belgium). μCT scanning was performed as follows: source voltage, 130 kV; source current, 60 μA; rotation step, 0.5°; exposure, 500 ms; image-pixel size, 13.85 μm. The obtained μCT-image data were reconstructed using NRecon software (v1.6.10.4, Bruker-CT). To prepare histological sections, tissue samples were processed by Technovit’s (Heraeus JULZER, Wehrheim, Germany) kit and equipment and stained.

### 4.7. Statistical Analysis

SPSS software, v20.0 (SPSS, Inc., Chicago, IL, USA) was used. Values are reported as the mean ± standard deviation (SD). Statistical differences were assessed using a non-parametric one-way Kruskal–Wallis test. *p* < 0.05 was considered to indicate statistical significance.

## 5. Conclusions

The osteogenic effect of combination bdECM+β-TCP treatment with PCL scaffold was better than when each was used separately. In the rat calvarial-defect model, the osteogenic effect was therapeutic when combined with a PCL scaffold. When the PCL scaffold structurally supported the defective bone tissue, bdECM and β-TCP gave rise to endogenous progenitor cells, which migrated to the defect area and differentiated. Furthermore, inflammation was lowest in the combination group, in vivo. Although the molecular mechanism was only confirmed in vitro, the clinical benefits improved when it was implanted and when the surroundings of the PCL scaffold were treated with bdECM+β-TCP mixture. Mixed bdECM+β-TCP are ideal bone-grafting materials because they promote bone formation with reduced inflammatory responses. Although the strengths of the materials studied here are weaker than those of metallic materials, these materials make postoperative monitoring easier, render removal surgery unnecessary, and produce forms similar to the original bone tissue.

## Data Availability

All results generated or analyzed during the present study are included in this published article. Data and materials will be made available upon request via email to first author (yuntobi@kpu.ac.kr).

## Supplementary Materials

The following are available online at https://www.mdpi.com/article/10.3390/ijms22169084/s1.
