# Supplementary material for: Bone Fracture-Treatment Method: Fixing 3D-Printed Polycaprolactone Scaffolds with Hydrogel Type Bone-Derived Extracellular Matrix and β-Tricalcium Phosphate as an Osteogenic Promoter
Source: Int J Mol Sci. 2021 Aug 23;22(16):9084. doi: 10.3390/ijms22169084 (PMC8396563; doi:10.3390/ijms22169084)
Supplement: Supplementary file 1 [file ijms-22-09084-s001.zip › ijms-1328244-supplementary.pdf]

International Journal of
Molecular Sciences

MDPI

Article

Bone Fracture-Treatment Method: Fixing 3D-Printed Polycaprolactone Scaffolds with Hydrogel Type Bone-Derived Extracellular Matrix and β-Tricalcium Phosphate as an Osteogenic Promoter

Seokhwan Yun [1], Dami Choi [2], Dong-Jin Choi [1], Songwan Jin [1,2], Won-Soo Yun [1,2,*,†], Jung-Bo Huh [3,*,†] and Jin-Hyung Shim [1,2,*,†]

1 Department of Mechanical Engineering, Korea Polytechnic University, Siheung-si 15073, Korea; yuntobi@kpu.ac.kr (S.Y.); seaottersarecute@protonmail.com (D.-J.C.); songwan@kpu.ac.kr (S.J.)
2 Research Institute, T&R Biofab Co., Ltd., Siheung-si 15073, Korea; choidm@tnrbiofab.com
3 Department of Prosthodontics, Dental Research Institute, Dental and Life Sciences Institute, School of Dentistry, Pusan National University, Yangsan-si 50612, Korea
* Correspondence: wsyun@kpu.ac.kr (W.-S.Y.); neoplasia96@daum.net (J.-B.H.); happyshim@kpu.ac.kr (J.-H.S.); Tel.: +82-31-8041-1819 (W.-S.Y.); +82-55-360-5146 (J.B.H.); +82-31-8041-1819 (J.-H.S.)
† Co-corresponding authors contributed equally to this work.

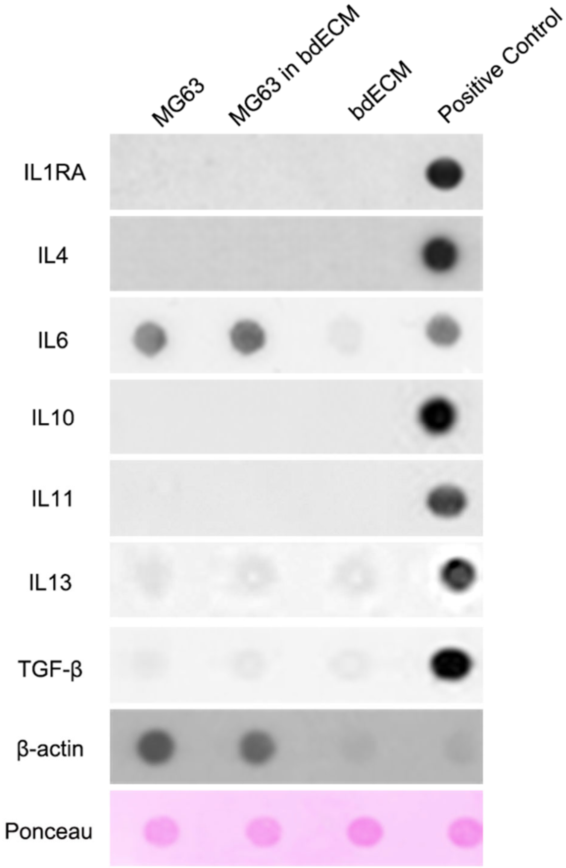

Figure S1. Expression of anti-inflammatory cytokines. IL1RA, IL4, Il6, IL10, IL11, IL13, TGF-β were immune blotted. Only IL-6 was detected in MG63 and MG63 in bdECM group and bdECM showed no IL6.

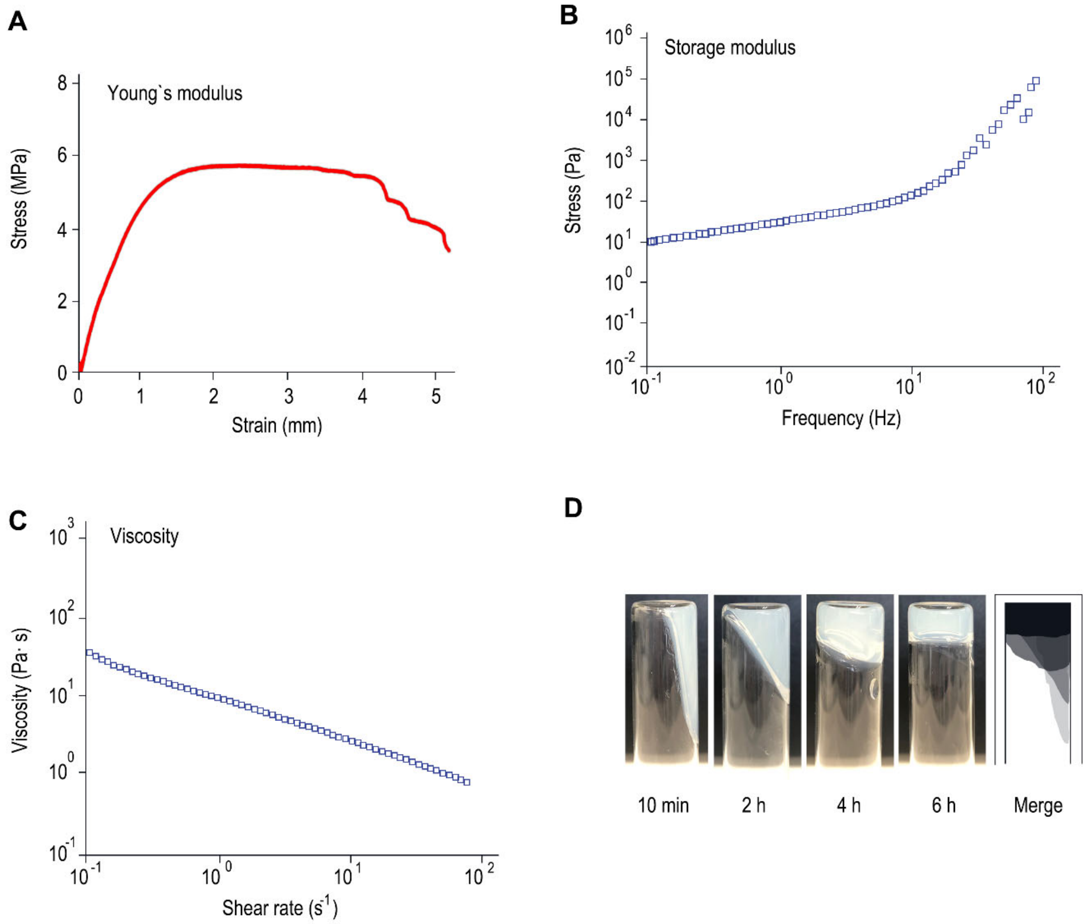

Figure S2. Mechanical properties of PCL and bdECM. (**A**) Young`s modulus test of PCL scaffold. (**B**) Storage modulus versus frequency graph of bdECM. (**C**) Stress-strain curve of PCL. (**D**) Flow by gravity showed viscosity of bdECM.

Table S1. Antibodies used for western blotting and immunohistochemistry.

Target protein	Host	Manufacturer	Domicilli
β-catenin	Rabbit	Cell Signaling	Danvers, MA, USA
p-β-catenin	Rabbit		
Smad	Rabbit		
p-Smad1/5	Rabbit		
Caspase-3	Rabbit		
IL-6	Rabbit		
β-actin	mouse	Sigma-Aldrich	St.Louis, MO, USA
Osteocalcin		Abcam	Cambridge, UK
MRP-14			
